# *Fraxinus mandshurica SPL2* enhances tolerance to drought stress by activating the phenylpropanoid pathway

**DOI:** 10.48130/forres-0026-0015

**Published:** 2026-04-14

**Authors:** Shuai Yang, Liang Gao, Shikang Zhao, Jialin Yan, Liming He, Biying He, Yaguang Zhan, Ying Xin, Fansuo Zeng

**Affiliations:** 1 State Key Laboratory of Tree Genetics and Breeding, Northeast Forestry University, Harbin 150040, China; 2 College of Life Science, Northeast Forestry University, Harbin 150040, China; 3 College of Forestry, Northeast Forestry University, Harbin 150040, China

**Keywords:** Fraxinus mandshurica, Transcriptome, Lignin biosynthesis, Phenylpropanoid, Drought stress

## Abstract

Drought is a major abiotic stressor that severely threatens forest tree growth and survival worldwide. The SQUAMOSA promoter binding protein-like (SPL) family is a group of plant-specific transcription factors that play important roles in plant growth, development, and environmental adaptation. Our study showed that overexpression of *Fraxinus mandshurica* *SPL2* (*FmSPL2*) promotes lignin biosynthesis and enhances drought tolerance. Phenotypic analysis indicated that the stomatal aperture of *FmSPL2*-OE1 and *FmSPL2*-OE2 was reduced by 12.05% and 19.23%, respectively, compared to the wild type (WT). Additionally, the xylem width of the sixth internode in these lines increased by 26.07% and 25.93% relative to WT. Correspondingly, lignin content was elevated by 19.87% and 18.48% compared with WT, respectively. Under drought stress, the lignin content in *FmSPL2*-OE plants remained elevated compared to WT. Furthermore*,* the overexpression lines exhibited increased SOD and POD activities, accompanied by reduced levels of MDA, H_2_O_2_, and superoxide anion. Transcriptome analysis indicated significant upregulation of multiple genes involved in phenylpropanoid biosynthesis in *FmSPL2*-OE plants, including *phenylalanine ammonia-lyase* (*PAL*), *cinnamyl alcohol dehydrogenase* (*CAD*), and *4-coumarate-CoA ligase* (*4CL*). Moreover, the yeast one-hybrid and dual-luciferase reporter assays demonstrated that FmSPL2 directly binds to the promoter regions of phenylpropanoid-related genes like *PAL*, *CAD*, and *PRX*, thereby activating the expression of these genes and enhancing lignin biosynthesis. These results elucidate a novel function of *FmSPL2* in enhancing drought tolerance through the regulation of phenylpropanoid pathway-mediated lignin accumulation.

## Introduction

Drought is a major environmental stressor that profoundly influences the growth conditions of trees as well as the structural and functional dynamics of ecosystems, thereby substantially affecting forestry development^[1]^. Plants have evolved a series of adaptive strategies to cope with drought through diverse physiological and morphological changes^[2]^. In plants, drought stress can damage the structural integrity of the xylem, resulting in hydraulic dysfunction and a reduction in water transport capacity. This stress decreases water content, induces stomatal closure, suppresses transpiration, and causes wilting, all of which collectively impair plant productivity. Additionally, it facilitates the generation of reactive oxygen species (ROS), which may lead to growth inhibition or cellular apoptosis^[3,4]^.

Lignin, a polyphenolic polymer ubiquitously found in vascular plants, is synthesized through the dehydrogenative polymerization of three primary monolignols: coumaryl alcohol, coniferyl alcohol, and sinapyl alcohol^[5]^. The genes central to lignin biosynthesis are critically involved in plant adaptive responses to drought stress. Studies have shown that drought conditions significantly upregulate the expression of the *PAL* gene, thereby facilitating lignin accumulation and enhancing plant stress resistance^[6]^. Similarly, the expression of *C4H* and *CAD* genes is also induced by drought, accompanied by an increase in lignin content. The newly synthesized lignin crosslinks with cellulose to form a rigid structure, promoting lignification and enhancing mechanical strength, which is essential for maintaining water transport and distribution^[7]^. Meanwhile, lignin monomers such as coniferyl alcohol and sinapyl alcohol exhibit antioxidant properties and can directly scavenge ROS^[8]^. Therefore, manipulating lignin biosynthesis presents a promising strategy to enhance plant drought tolerance. Increased expression of lignin biosynthetic enzymes has been associated with improved hydraulic conductivity and enhanced adaptation to drought stress^[9,10]^.

Multiple families of transcription factors (TFs), such as NAC (NAM, ATAF, and CUC), MYB, WRKY, bZIP (basic leucine zipper), and AP2/ERF(APETALA2/ETHYLENE RESPONSIVEFACTOR), have been extensively reported to participate in the regulation of drought tolerance^[11−13]^. Among these, SPL proteins represent a unique category of plant TFs that are critically involved in modulating plant development, as well as responses to abiotic stress^[14]^. The SPL transcription factor contains a highly conserved SBP domain of approximately 76 amino acid residues. This domain consists of two tandem zinc fingers (Cys-Cys-His-Cys and Cys-Cys-His) and a nuclear localization signal at the C-terminus, and regulates gene expression by binding to the GTAC sequence^[15]^. In *Arabidopsis thaliana*, *AtSPL7* plays a critical role in mediating responses to copper deficiency under high nitrogen conditions^[16]^. The transcription factor BZR1 promotes vegetative phase transition by regulating the activity of *AtSPL9*^[17]^, and the DELLA protein influences axillary bud formation by interacting with *AtSPL9*^[18]^. In wheat studies, *TaSPL14* has been found to affect plant height, panicle length, spikelet number, and 1,000-grain weight, and may regulate wheat spike development through the ethylene response pathway. *TaSPL16* can delay vegetative leaf development, and promote early flowering, increase organ size, and affect yield-related traits^[19,20]^. In *Lilium*, overexpression of *LbrSPL9* and *LbrSPL15* has been reported to accelerate flowering^[21]^. Studies on *Salvia miltiorrhiza* reveal that *SmSPL6* not only regulates root development, but also enhances phenolic acid accumulation while repressing anthocyanin biosynthesis^[22]^. Conversely, *SmSPL7* negatively regulates phenolic acid accumulation and modulates anthocyanin content in salvia^[23]^. In rice research, *OsSPL9* has been identified as a regulator of grain number and yield; overexpression of *OsSPL12* enhanced seed dormancy, and *OsSPL10* can regulate rice trichome formation also affecting ROS production through direct regulation of *OsNAC2* transcription, which in turn regulates drought resistance^[24−27]^. Crucially, recent research has elucidated the involvement of SPLs in secondary cell wall biosynthesis and xylem development. For instance, in *Panicum virgatum*, PvSPL2 directly binds to the promoters of lignin biosynthetic genes, such as *CCR1*, *F5H*, and *COMT*, thereby regulating lignin accumulation^[28]^. Similarly, overexpression of *OsSPL14* in rice significantly upregulates key lignin biosynthetic genes including *PAL*, *C4H*, and *4CL*, resulting in increased lignin content and enhanced stem mechanical strength^[29]^. Furthermore, in the woody plant *Jatropha curcas*, overexpression of *rJcSPL9* has been shown to promote xylem development^[30]^. Collectively, these findings underscore the pivotal roles of *SPL* genes as key regulators of diverse plant processes, encompassing seed size, dormancy, flowering time, phase transitions, abiotic stress responses, xylem development, hormone signaling, and phenylpropanoid biosynthesis. Nevertheless, the functions of *SPL* genes in mediating drought stress responses in woody plants remain underexplored.

*Fraxinus mandshurica*, with its graceful shape, intricate texture, and superior wood quality, exhibits resilience to cold temperatures and pest infestations, making it a premium material for both industrial and civilian applications, with significant economic value^[31]^. Additionally, it serves as a primary constituent species in the forests of Northeast China, playing a crucial ecological role in water conservation and the prevention of soil erosion^[32]^. Throughout its life cycle, *Fraxinus mandshurica* often faces water shortages, which significantly affect the development of both primary and secondary cell walls, thus significantly affecting wood formation. Consequently, gaining insight into the molecular mechanisms behind its drought tolerance is important for selecting and cultivating drought-tolerant varieties.

Our earlier research showed that *FmSPL2* regulates leaf size, root development, and flowering time, and responds to both drought and salt stress^[33]^. However, the detailed molecular mechanisms by which *FmSPL2* contributes to drought adaptation and xylem development are not well understood. In this study, we investigate the function of *FmSPL2* in xylem development and drought tolerance, revealing that it enhances drought resistance by directly regulating the expression of key enzymes in the phenylpropanoid metabolism pathway, thereby promoting lignin synthesis. This study elucidates the molecular mechanisms of *FmSPL2*-mediated drought stress response and xylem development, laying the groundwork for genetic improvement of *Fraxinus mandshurica*, with significant potential to enhance wood quality and stress resilience.

## Materials and methods

### Plant materials and growth conditions

Our research team previously cloned the *FmSPL2* gene to obtain transgenic tobacco lines, *FmSPL2*-OE1 and *FmSPL2*-OE2, which were the fourth-generation transgenic materials^[33]^. The exogenous gene is a single copy and integrated. Both the *FmSPL2* transgenic and wild-type tobacco were transplanted to a 3:1 ratio of charcoal to vermiculite in a nutrient soil. The growth conditions included 16 h of light followed by 8 h of darkness, a temperature of 22 ± 3 °C, and a relative humidity of 60%−75%. Seedlings with consistent growth were selected for subsequent stress treatments.

One-year-old *Fraxinus mandshurica* seedlings that were growing uniformly were chosen and transplanted into 10 cm pots filled with a soil and perlite mixture in a 3:1 ratio for cultivation. All experiments took place in a greenhouse under a 16-h light and 8-h dark cycle, with temperatures maintained at 25 ± 3 °C and relative humidity at 60% ± 10%. The VIGS experiments were performed during the active growing period of the seedlings.

### VIGS-mediated *FmSPL2* silencing

In June, during the active growth stage of *Fraxinus mandshurica*, virus-induced gene silencing (VIGS) was performed. The VIGS procedure employed the pTRV1 and pTRV2 vectors derived from tobacco rattle virus (TRV). To reduce off-target silencing, a gene-specific fragment of 300 base pairs targeting *FmSPL2* was designed. The primers used for PCR amplification were engineered to overlap by 15 base pairs with the linearized pTRV2 vector and incorporated a restriction enzyme recognition site. The pTRV1 + pTRV2-*FmSPL2* construct was introduced into the stems and leaves of *Fraxinus mandshurica*, while samples transformed with the empty pTRV2 vector served as a negative control (pTRV1 + pTRV2). After a 2-d incubation period in darkness, the cultures were exposed to light for 12 d. Total DNA and RNA were then extracted for further analysis. Virus accumulation and gene silencing efficiency were assessed using PCR and quantitative reverse transcription PCR (qRT-PCR). The primers used for cloning, PCR, and qRT-PCR are listed in Supplementary Table S1.

### *FmSPL2* transgenic tobacco phenotype analysis under drought stress

We subjected transgenic tobacco plants to both controlled water drought and simulated drought treatments. WT and *FmSPL2*-OE tobacco seeds were germinated on a resistant medium containing 20 mg/L hygromycin B. Once the seedlings reached similar growth stages, they were transplanted into culture bowls measuring 100 mm in diameter and 85 mm in height. After one week of normal growth, water-controlled treatments were applied for 0, 5, and 10 d, and the phenotype was recorded with a camera. For simulated drought, 200 and 300 mM mannitol were added to MS medium, and plants were cultured for 20 d, during which the phenotype was recorded with a camera. Additionally, WT and *FmSPL2*-OE tobacco plants were grown in soil under water-controlled natural drought conditions, with a normally watered group as the control. The phenotypes were recorded at 0, 10, 20, 30, and 40 d, respectively.

### Xylem staining and lignin content determination

0.5 g of phloroglucinol powder was dissolved in 10 mL of 95% alcohol to form a phloroglucinol-alcohol solution. The drought-treated wild-type and transgenic *FmSPL2* tobacco was used as the experimental group, and non-drought-treated tobacco plants were used as the control group. Two blades were stacked together, and the stem segment cross-sections of the third and sixth segments of the plant were removed from top to bottom and placed on a glass slide. A drop of concentrated hydrochloric acid, and then a drop of phloroglucinol-alcohol solution was added, the glass slide covered with a coverslip and then imaged with a microscope.

Control and experimental group samples were first dried, ground, and passed through an 80-mesh sieve. Subsequently, 0.05 g of the resulting powder was dissolved in 1 mL of 25% bromoacetic acid in glacial acetic acid. The mixture was then incubated in a 70 °C water bath for 30 min. Following incubation, the reaction was terminated by adding 0.4 mL of 2 mol/L NaOH. Next, 1 mL of glacial acetic acid and 0.04 mL of 0.5 mol/L hydroxylamine hydrochloride were added. The solution was centrifuged at 6,000 rpm for 10 min. The supernatant was then diluted for measurement (100 μL of extract was brought to a final volume of 5 mL with distilled water). The lignin content was indicated by measuring the OD value at 280 nm, with the experiment repeated four times. Lignin content (OD/g/FW) was calculated as: absorption value × 100/(absorption coefficient × sample concentration [g/L]), where the absorption coefficient is 20.2 L/(g·cm).

### Stomatal change and determination of chlorophyll fluorescence

At 12:00 noon, the leaves of the *FmSPL2* transgenic and WT tobacco plants before and after drought treatment, were stripped of the dorsal epidermis of the leaves with forceps, placed on a glass slide, covered with a coverslip, and observed under a microscope. The stomata observed through the imaging microscope were measured directly by computer for stomatal opening width, and the number of stomata was counted in a fixed field of view and later recorded. Additionally, chlorophyll fluorescence parameters were measured using a chlorophyll fluorescence imager with default settings, and the values of F_0_, F_m_, and F_v_ were recorded.

### DAB and NBT staining, determination of antioxidant indexes

The treated leaves were stained with 3,3-diaminobenzidine (DAB) for 12 h and with nitroblue tetrazolium (NBT) for 4 h. After staining, the leaves were boiled in 95% alcohol to decolorize, and the leaves were photographed after the chlorophyll was completely removed.

The activities of SOD and POD, along with the MDA content in the samples were measured using specialized assay kits (Beijing Solarbio Science & Technology Co., Ltd., Beijing, China) according to the manufacturer's guidelines. The plants with uniform growth after treatment, and the plants without treatment as the control were selected, with three replicates for each variable. Consistent with the above selection, the H_2_O_2_ content was measured using an H_2_O_2_ detection kit, and the superoxide anion content was determined using a superoxide anion detection kit.

### Germination rate and pollen vigor determination

WT and *FmSPL2*-OE seeds were germinated on 1/2 MS medium as the control group, 1/2 MS medium containing 200 mM and 300 mM mannitol as experimental groups, and the germination rate of seeds was recorded.

Treated and untreated fresh flowers were collected, and the petals and pistils were carefully removed. The pollen was placed on a glass slide, and 2−3 drops of Alexander staining solution were added. The mixture was thoroughly mixed and immediately covered with coverslips for 5−10 min staining. Excess liquid is apsirated, and observation is under a microscope.

### *Fraxinus mandshurica* transient infection and quantitative detection

*Fraxinus mandshurica* seedlings and bacterial suspension were prepared in advance. On an ultra-clean bench, sterile *Fraxinus mandshurica* seedlings were soaked in a hypertonic solution (WPM solution containing 25% sucrose) for 15 min. The infection solution was made from WPM liquid suspension, and the *Fraxinus mandshurica* seedlings were moved into the transformed liquid, sonicated for 90 s, vacuum-infiltrated for 15 min, and co-cultured for 2.5 h. Afterward, the seedlings were blotted dry on filter paper and placed on solid WPM medium for dark culture for 3 d. It was then soaked in WPM solution containing 20% PEG6000 for 0, 1, 3, 6, and 12 h, respectively, and then stored at −80 °C.

The *Fraxinus mandshurica* seedlings that were not infected served as the control group, while the infected seedlings comprised the experimental group. Both the experimental and the control groups of *Fraxinus mandshurica* seedlings were subjected to liquid nitrogen mortar. RNA was then extracted from the samples using the Tris-CTAB method, followed by reverse transcription of the RNA. For quantitative analysis, the *Fraxinus mandshurica* actin gene (*Fmactin*) was selected as the internal reference gene (Supplementary Table S1).

### Transcriptome sequencing

After drought treatment, three control tobacco plants and three transgenic tobacco plants from the same batch and exhibiting similar growth status were selected. For each plant, 0.5 g of leaf tissue was wrapped in tin foil and quickly frozen in liquid nitrogen. All samples were stored on dry ice and sent to Beijing Biomarker Technologies for high-throughput sequencing using the Illumina platform, followed by bioinformatics analysis. The three controls were named GH-WT-1, GH-WT-2, and GH-WT-3, and the three transgenic lines were named GH-919-1, GH-919-2, and GH-919-3.

Total RNA was extracted from all samples using the TRIzol method, the purity of RNA samples was tested by equipment to ensure the quality of the samples, and the library was then constructed. Firstly, the eukaryotic mRNA was enriched by magnetic beads with Oligo (dT), and then the mRNA was randomly interrupted by a fragmentation buffer. The first cDNA strand was then synthesized using six-base random primers with mRNA as template, followed by the addition of buffer, dNTPs, RNase H, and DNA polymerase I to synthesize the second cDNA strand and purify the cDNA using AMPure XP beads. After that, the purified double-stranded cDNA was end-repaired, a-tailed, and ligated with a sequencing linker, then the fragment size was selected with AMPure XP beads, and the cDNA library was enriched by PCR. After the construction of the library, qPCR was used to accurately quantify the effective concentration of the library (the effective concentration of the library > 2 nM) to ensure the quality of the library. After the library was certified, the different libraries were pooled according to the target amount of off-line data, and sequenced with the Illumina platform.

### Yeast one-hybrid

According to the experimental design, the constructed pGADT7 (AD)-*FmSPL2* vector was co-transformed with the pHIS2 promoter sequence vector into yeast competent Y1H strains. Verified positive strains were cultured overnight in DDO (double dropout) liquid medium at 30 °C for amplification. After centrifugation, the yeast cells were collected, resuspended in 0.9% NaCl, and adjusted to OD_600_ = 0.5. This suspension was then evenly spread onto TDO (SD/His/-Leu/-Trp) and TDO/3-AT (90 mg/L) media. The plates were incubated at 30 °C for 3–5 d. Colony growth was observed and photographed for record-keeping.

### Dual-luciferase reporter gene assay

The constructs pGreenII62-SK-*FmSPL2* and pGreenII0800-LUC-*proFmPAL1*, *proFm4CL1*, *proFm4CL2*, *proFmCCR1*, *proFmHCT1*, *proFmHCT2*, *proFmCAD1*, *proFmCAD2*, *proFmPRX1*, *proFmPRX2*, *proFmPRX3*, and *proFmCOMT1* were individually transformed into *Agrobacterium tumefaciens* strain GV3101 (pSoup). The verified colonies were cultured overnight at 28 °C, harvested by centrifugation, and resuspended in an infiltration buffer (10 mM MgCl_2_, 10 mM MES, and 150 μM acetosyringone). The suspensions were incubated in the dark at room temperature. Mixed suspensions were infiltrated into *N. benthamiana* leaves. After 1 d dark and 2 d light incubation, luminescence images were captured. Luciferase activities were measured using a Double-Luciferase Reporter Assay Kit (GeneCreate Biological Engineering Co., Ltd., Wuhan, China) following the manufacturer's instructions. The LUC : REN ratio uses 35S as the control and is set to 1 for normalization.

### Statistical analysis and data processing

Statistical significance was determined using a one-way Student's *t*-test (two-tailed) performed in SPSS software (v.26.0), with significance defined as * *p* < 0.05 and ** *p* < 0.01, and the data was normalized, showing normal distribution and homogeneity of variance.

## Results

### *FmSPL2* is involved in the response to drought stress

**Figure 1:**
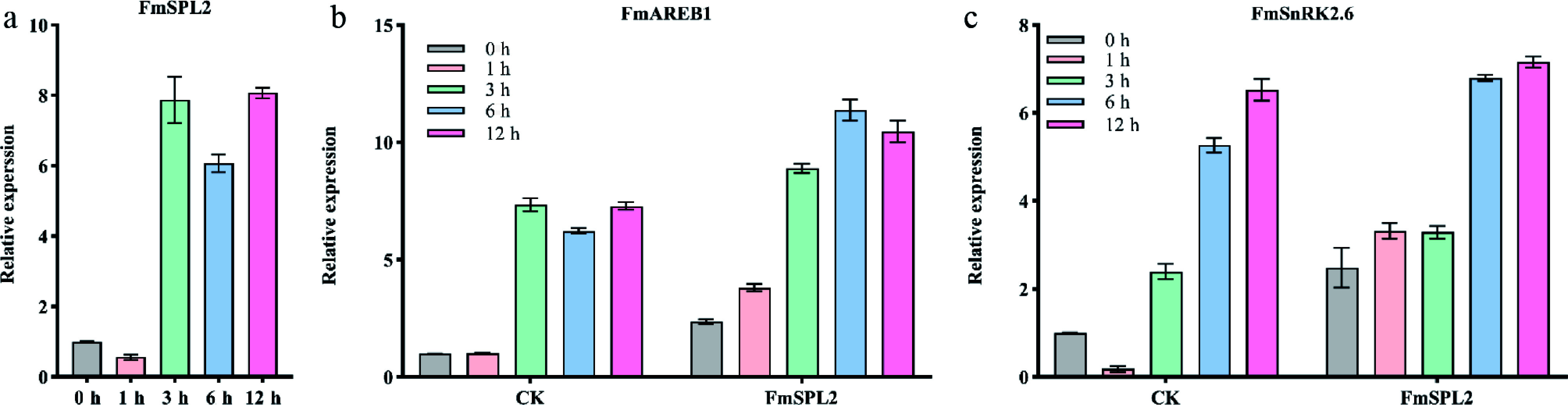
The expression of the *FmSPL2* gene is regulated by drought stress. (a) The expression of *FmSPL2* under drought treatment at 0, 1, 3, 6, and 12 h. (b) The expression changes of *FmAREB1* after transient transfection of *FmSPL2* and natural drought treatment at 0, 1, 3, 6, and 12 h. (c) The expression levels of *FmSnRK2.6* after transient transfection of *FmSPL2* and drought treatment at 0, 1, 3, 6, and 12 h.

Previous research has shown that *FmSPL2* responds to abiotic stress^[33]^. Therefore, we subjected *Fraxinus mandshurica* seedlings to a 3-h simulated drought treatment using 20% PEG6000 and observed a 6.1 to 8.0-fold upregulation in the expression of the *FmSPL2* gene, suggesting that *FmSPL2* plays a role in drought stress induction. Subsequently, we transiently transfected the *FmSPL2* gene into *Fraxinus mandshurica* seedlings and subjected them to simulated drought stress by immersing them in 20% PEG6000. Previous research has indicated that *SnRK2.6* and *AREB1* are key regulators in enhancing plant tolerance to drought stress. Transcriptomic analysis showed that in the control group, the expression of *SnRK2.6* and *AREB1* increased after drought treatment. Notably, in seedlings transiently expressing *FmSPL2*, *SnRK2.6* expression began to increase 1 h after drought treatment, while *AREB1* expression also increased, peaking after 6 h of drought stress (Fig. 1b, c). These findings provide preliminary evidence that *FmSPL2* may facilitate the expression of drought-responsive genes in plants, thereby contributing to enhanced drought tolerance.

### VIGS-induced silencing of *FmSPL2* reduces the drought tolerance of *Fraxinus mandshurica*

**Figure 2:**
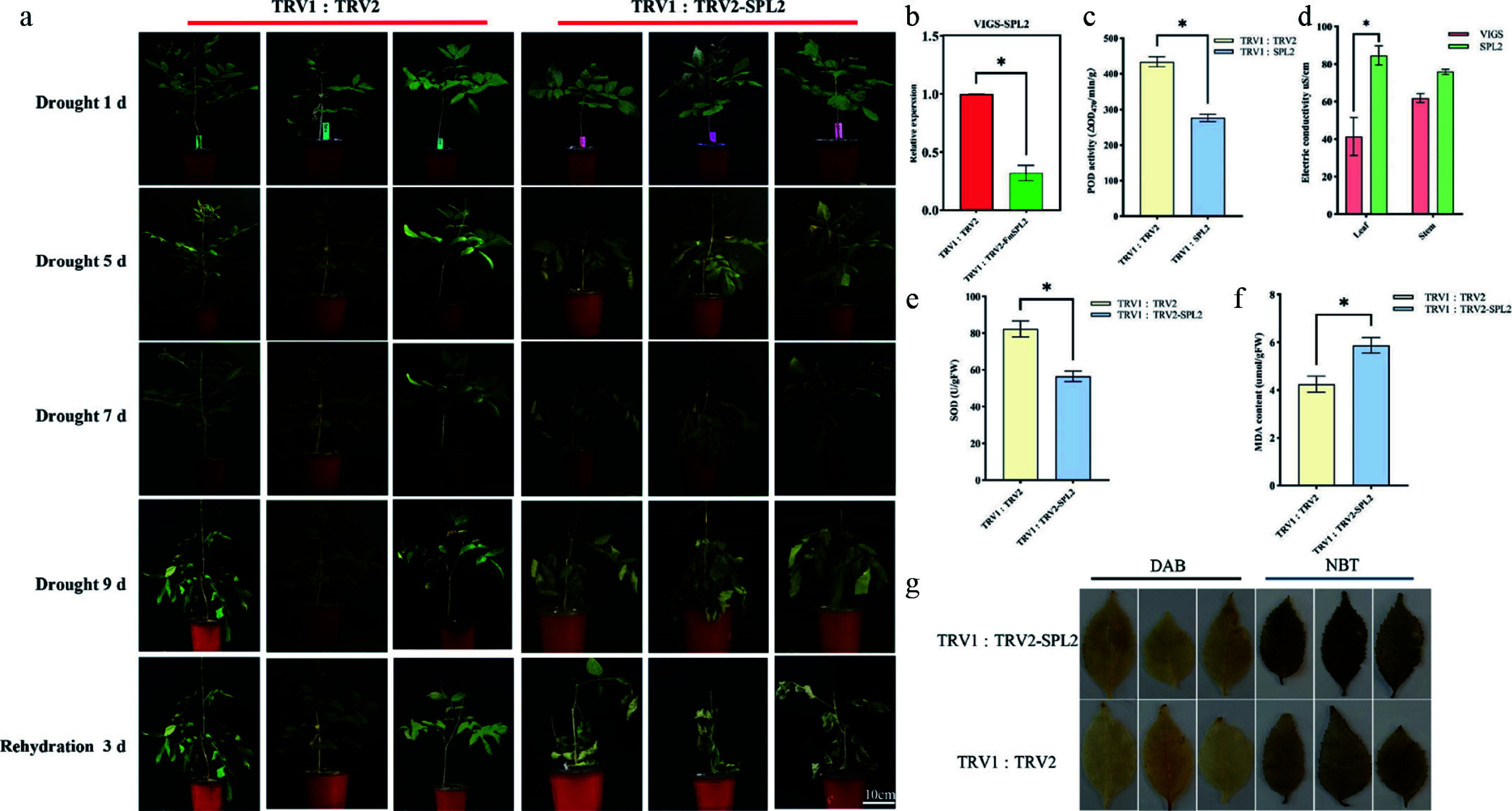
Phenotypic analysis of *FmSPL2* gene silencing by VIGS in *Fraxinus mandshurica*. (a) VIGS-*FmSPL2* drought phenotype analysis; (b) VIGS-*FmSPL2* qPCR analysis; (c) POD activity assay; (d) electrical conductivity measurement; (e) SOD activity assay; (f) MDA content assay; (g) DAB and NBT staining.

Virus-induced gene silencing (VIGS) was employed to silence the *FmSPL2* gene in *Fraxinus mandshurica* by constructing pTRV2-*FmSPL2*. Fourteen days after inoculation, with pTRV1 : pTRV2 empty vector plants as controls, qRT-PCR analysis showed that *FmSPL2* expression in the silenced plants dropped to 31.85% of the control level (Fig. 2b). After 9 d of natural drought, both groups exhibited leaf wilting, but the pTRV2-*FmSPL2* plants displayed more severe wilting (Fig. 2a). Physiological measures revealed that the activities of POD and SOD in the pTRV2-*FmSPL2* group decreased by 36.3% and 31.3%, respectively, while the relative conductivity in the leaves and stems increased by 104.5% and 22.3% (Fig. 2c–e). Additionally, MDA content, which indicates lipid peroxidation levels, increased by 38.6% in the pTRV2-*FmSPL2* plants after drought stress (Fig. 2f). Similarly, DAB and NBT staining results indicated a reduction in drought resistance in the pTRV2-*FmSPL2* plants (Fig. 2g).

### Phenotypic analysis of *FmSPL2* transgenic tobacco under drought stress

**Figure 3:**
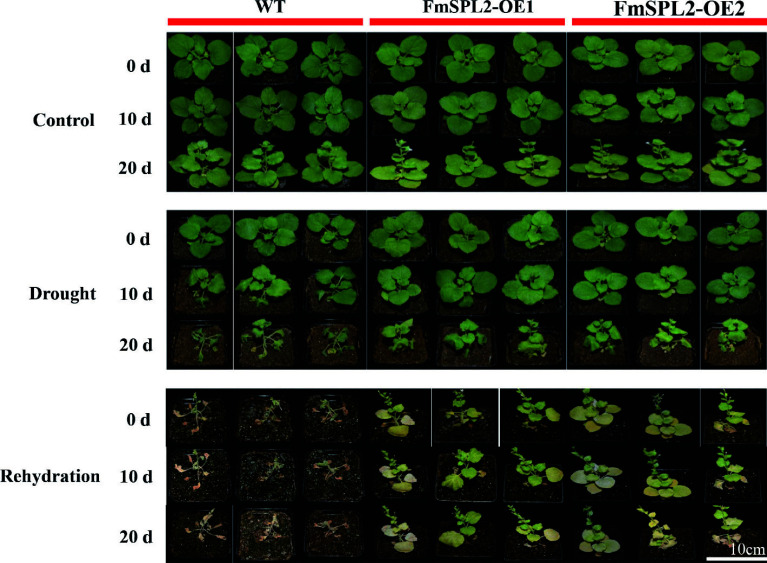
*FmSPL2* transgenic tobacco drought treatment phenotype image. Fifteen days of soil cultivation with drought stress and rehydration after stress, drought treatment for 20 d, followed by 15 d of rehydration.

Uniformly growing *FmSPL2* transgenic tobacco plants were subjected to drought stress on MS medium supplemented with 200 mM and 300 mM mannitol. The results demonstrated that root initiation was observed in the transgenic tobacco lines *FmSPL2*-OE1 and *FmSPL2*-OE2 on day 10 in the 200 mM mannitol MS medium, whereas the WT plants did not develop roots at this stage, and only began to form roots after 15 d of culture (Supplementary Fig. S1). In MS medium supplemented with 300 mM mannitol, transgenic tobacco lines *FmSPL2*-OE1 and *FmSPL2*-OE2 initiated root development by day 10 and continued to grow well in subsequent cultures. On the contrary, WT tobacco failed to develop roots even after 15 d (Supplementary Fig. S1). Moreover, in the soil culture experiment, WT tobacco leaves exhibited wilting after 10 d of drought stress compared to the control, while neither of the transgenic lines, *FmSPL2*-OE1 or *FmSPL2*-OE2 showed any wilting symptoms. Following 20 d of drought treatment, WT plants were severely withered, whereas the transgenic lines only manifested mild wilting (Fig. 3). In summary, the results from both the simulated and water-controlled drought experiments consistently indicate that *FmSPL2* transgenic tobacco exhibited enhanced drought tolerance. These results suggest that FmSPL2 plays a crucial role in regulating drought adaptation.

### *FmSPL2* influences drought resistance by affecting stomatal aperture and photosynthetic rate

**Figure 4:**
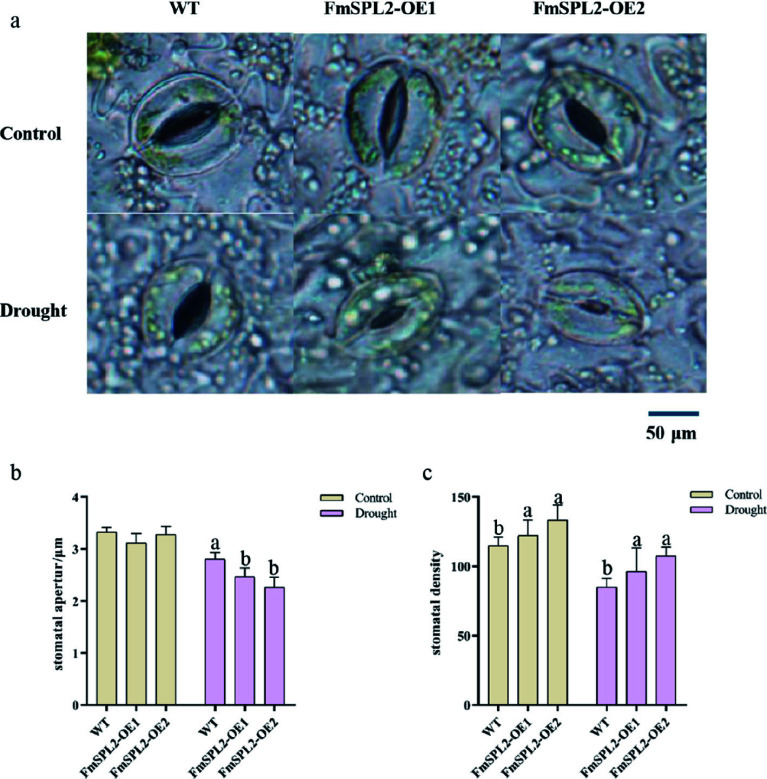
Effect of *FmSPL2* on stomata under drought treatment. (a) Stomatal state after drought treatment; (b) stomatal aperture; (c) stomatal density.

Drought stress induces a decrease in water content, stomatal closure, and suppressed photosynthetic rates in plants. To investigate the mechanisms conferring drought resistance in *FmSPL2* transgenic plants, we employed a microscopic observation of stomatal apertures and chlorophyll fluorescence imaging to assess chlorophyll fluorescence parameters. Our findings indicate that, following 15 d of drought treatment, stomatal size was significantly reduced in the leaves of *FmSPL2*-OE1 and *FmSPL2*-OE2 lines compared to the WT controls (Fig. 4a). After drought stress, the stomatal aperture of *FmSPL2*-OE1 and *FmSPL2*-OE2 decreased by 12.05% and 19.23%, respectively, relative to the WT (Fig. 4b). Moreover, stomatal density in *FmSPL2*-OE1 and *FmSPL2*-OE2 increased by 11.54% and 26.09%, respectively, compared to the WT (Fig. 4c). In summary, these alterations in stomatal aperture and density between transgenic and WT tobacco under drought stress suggest that the transgenic plants modulate leaf respiration to conserve water, thereby enhancing their drought tolerance.

**Figure 5:**
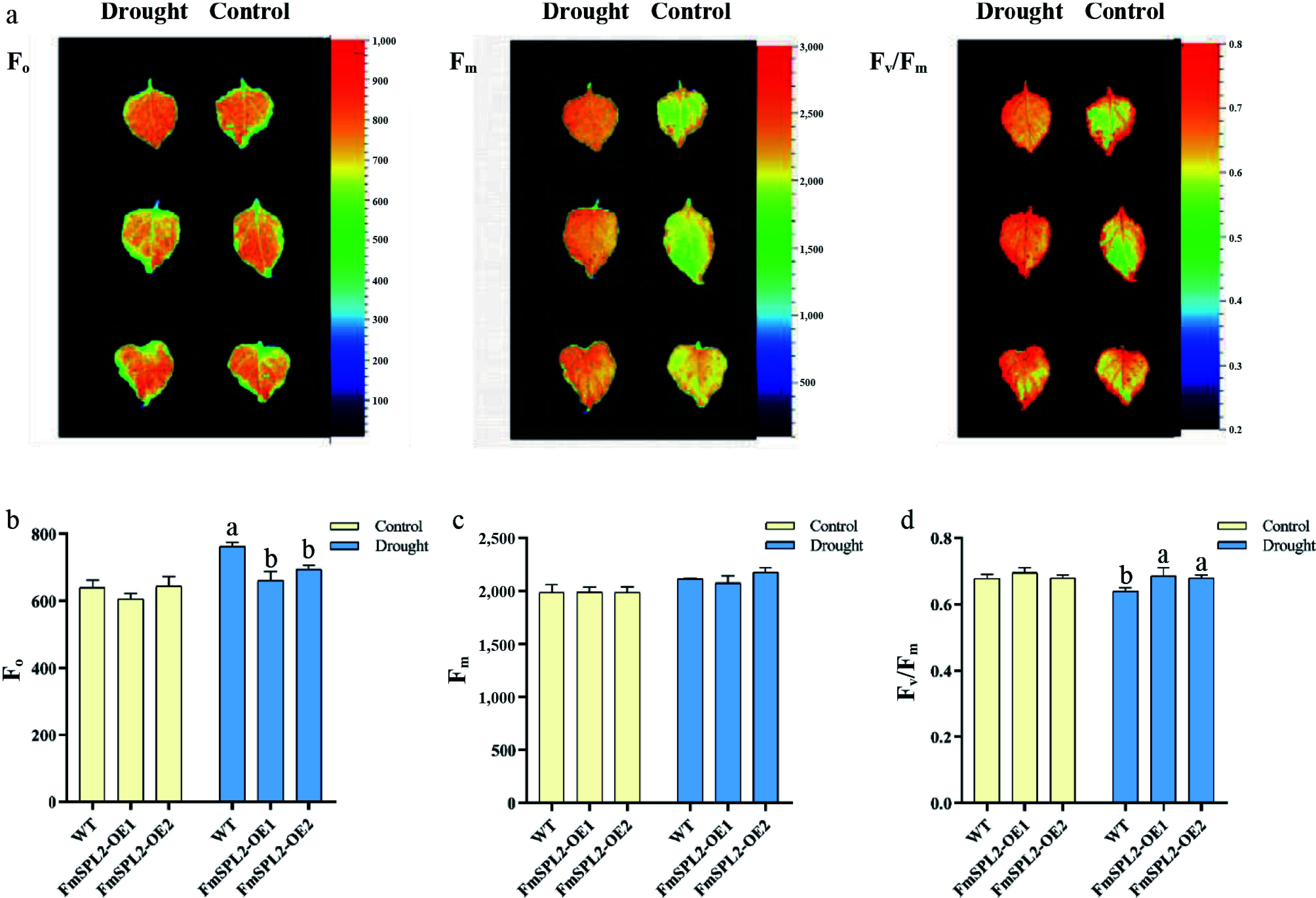
Changes of chlorophyll fluorescence parameters after drought stress. (a) Chlorophyll fluorescence imaging; (b) F_0_ value of chlorophyll fluorescence parameter; (c) chlorophyll fluorescence parameter F_m_ value; (d) chlorophyll fluorescence parameter F_v_/F_m_ value.

The chlorophyll fluorescence parameter F_0_ represents the minimal fluorescence yield, corresponding to the fluorescence when the photosystem II (PSII) reaction center is fully open. It is closely associated with chlorophyll concentration in the leaves. Conversely, F_m_ denotes the maximal fluorescence yield, observed when the PSII reaction center is fully closed. The parameter F_v_/F_m_ serves as an indicator of the maximum photochemical quantum efficiency of PSII. Following 15 d of drought treatment, F_0_ values in the *FmSPL2*-OE1 and *FmSPL2*-OE2 lines were reduced by 13.42% and 8.87%, respectively, relative to WT (Fig. 5b), whereas no significant changes were observed in F_m_ values (Fig. 5c). In contrast, the F_v_/F_m_ values in *FmSPL2*-OE1 and *FmSPL2*-OE2 increased by 7.29% and 6.25%, respectively, relative to WT following drought treatment (Fig. 5d). Under non-stress conditions, the F_v_/F_m_ values remained relatively stable and were unaffected by genotype or growth conditions. Collectively, these results provide further evidence that overexpression of the *FmSPL2* gene enhances the plant's drought stress adaptation capacity.

### *FmSPL2* can enhance drought tolerance by scavenging ROS

**Figure 6:**
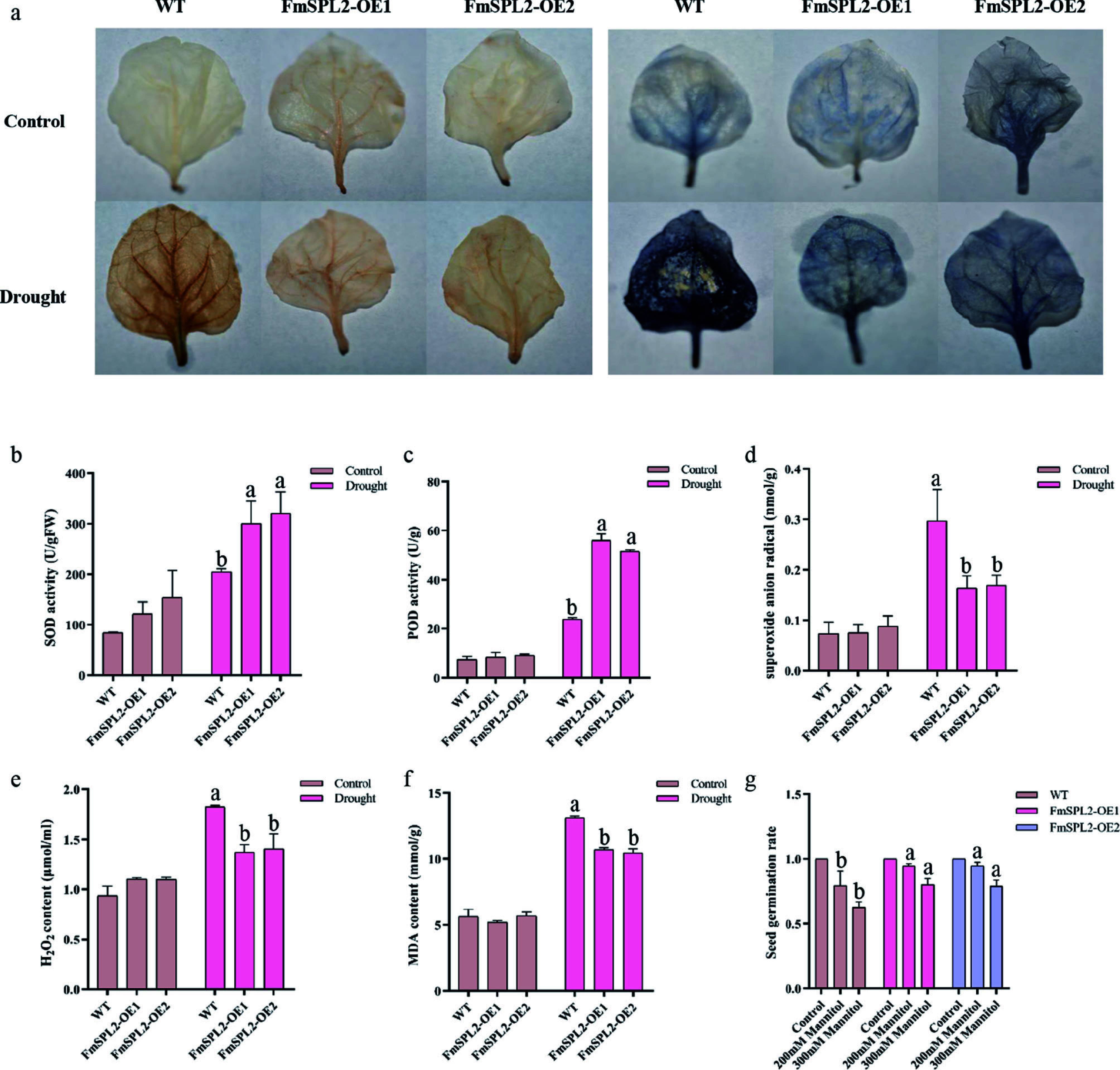
Physiological indexes determination after drought treatment. (a) Hydrogen peroxide (DAB), and superoxide anion (NBT) staining; (b) superoxide dismutase (SOD) activity; (c) peroxidases (POD) activity; (d) superoxide anion (DFR) content; (e) hydrogen peroxide (H_2_O_2_) content; (f) Malondialdehyde (MDA) content, and (g) germination rate.

When plants are exposed to abiotic stress, they generate excessive levels of ROS, including hydrogen peroxide (H_2_O_2_) and superoxide anions (O_2_^−^). These ROS can initiate lipid peroxidation in cellular membranes, with malondialdehyde (MDA) serving as a biomarker for membrane lipid peroxidation. Antioxidant enzymes, such as superoxide dismutase (SOD) and peroxidases (POD), are pivotal in mitigating the detrimental effects of ROS by scavenging O_2_^−^ and H_2_O_2_, thereby protecting cellular structures and maintaining plant integrity under stress conditions. Following 15 d of drought treatment, the leaves of *FmSPL2*-OE and WT plants were stained with 3,3'-Diaminobenzidine Tetrahydrochloride (DAB) for H_2_O_2_ detection, and Nitrotetrazolium Blue chloride (NBT) for O_2_^−^ detection. The *FmSPL2*-OE leaves displayed notably lighter staining patterns compared to WT plants (Fig. 6a), suggesting a reduced accumulation of H_2_O_2_ and superoxide anions in the transgenic plants under drought stress. Furthermore, the analysis of antioxidant enzyme activities revealed significant increases in SOD and POD activities in *FmSPL2*-OE plants following drought treatment. Specifically, SOD activity rose by 46.65% and 56.57%, while POD activity increased by 1.33-fold and 1.15-fold relative to WT (Fig. 6b, c). This upregulation of antioxidant enzyme activity corresponded with reductions in H_2_O_2_ and O_2_^−^ levels. In comparison to WT, H_2_O_2_ content in the transgenic plants was reduced by 24.96% and 23.15%, while O_2_^−^ levels decreased by 44.75% and 42.90%, respectively (Fig. 6d, e). Additionally, the MDA content, indicative of membrane lipid peroxidation, was lower in the *FmSPL2*-OE plants, with reductions of 18.41% and 20.38% relative to WT (Fig. 6f), suggesting attenuated membrane damage. To evaluate the potential trade-offs between growth and stress tolerance, seed germination rates were assessed under simulated drought conditions using MS medium supplemented with 200 mM and 300 mM mannitol. Although germination rates declined with increasing mannitol concentrations, *FmSPL2*-OE seeds exhibited significantly higher germination rates than WT seeds (Fig. 6g). In summary, these findings indicate that overexpression of *FmSPL2* enhances antioxidant enzyme activities, promotes ROS scavenging, reduces drought-induced cellular damage, and improves seed germination under drought stress, thereby conferring increased drought tolerance to the transgenic plants.

### *FmSPL2* positively regulates lignin biosynthesis

**Figure 7:**
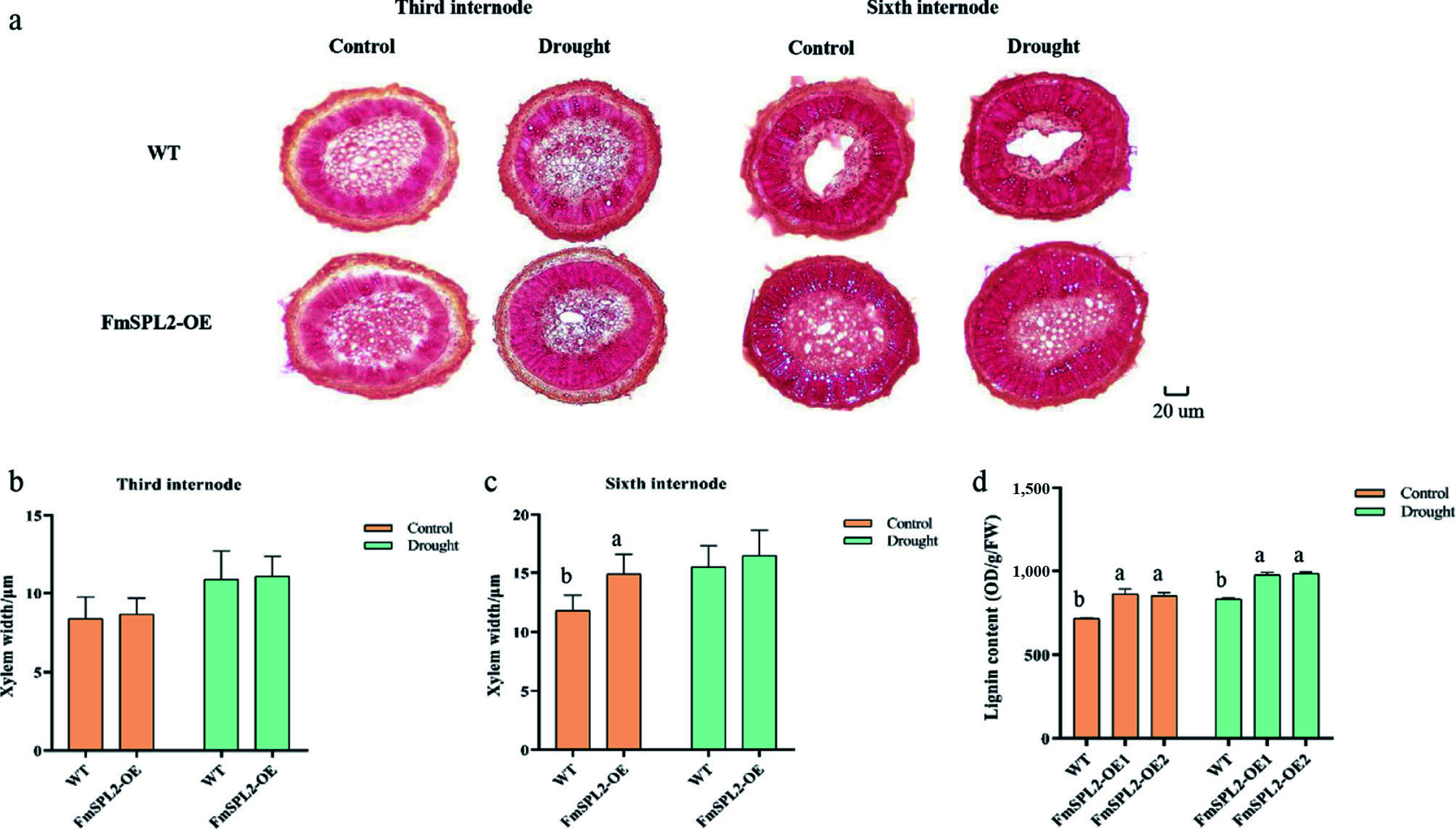
*FmSPL2* gene affects xylem development. (a) Xylem staining after drought treatment; (b) xylem thickness of the third internode; (c) xylem thickness of the sixth internode, and (d) lignin content.

The xylem of WT, *FmSPL2*-OE1, and *FmSPL2*-OE2 plants was stained in the third and sixth internodes following drought treatment. Under normal growth conditions, no significant differences in xylem width were observed between WT and *FmSPL2*-OE plants at the third internode. However, after 15 d of drought treatment, both WT and *FmSPL2*-OE plants exhibited xylem thickening (Fig. 7a, b). At the sixth internode, the xylem of *FmSPL2*-OE plants was 26% thicker than that of WT plants under normal conditions. Notably, after drought treatment, no statistically significant difference in xylem thickness was observed between *FmSPL2*-OE and WT plants (Fig. 7c). Moreover, lignin content analysis revealed that under control conditions, *FmSPL2*-OE1 and *FmSPL2*-OE2 showed increases of 19.87% and 18.48%, respectively, compared to WT. Following drought treatment, the lignin content of *FmSPL2*-OE1 and *FmSPL2*-OE2 increased by 17.23% and 18.26%, respectively, relative to WT (Fig. 7d). In summary, these findings indicate that *FmSPL2* transgenic tobacco exhibited higher xylem thickness and lignin content than WT tobacco, suggesting that *FmSPL2* improves drought resistance through the reinforcement of lignin biosynthesis.

### Transcriptomic analysis reveals differentially expressed genes in both *FmSPL2*-OE and WT plants after drought stress

**Figure 8:**
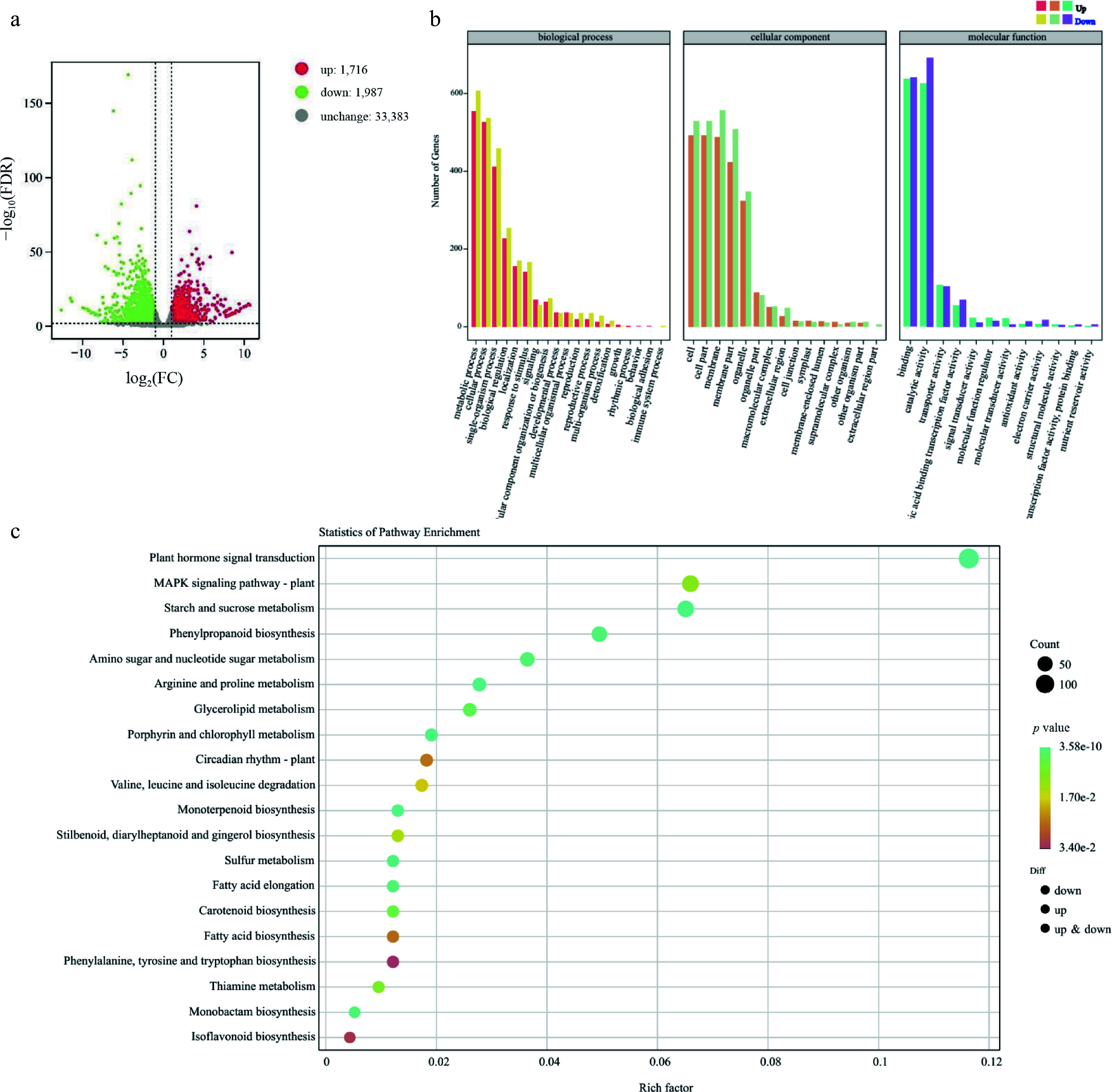
*FmSPL2* affects drought resistance by regulating the expression of lignin biosynthesis genes.

To investigate whether the *FmSPL2* regulatory network is involved in lignin biosynthesis and drought tolerance, we conducted a comparative transcriptomic analysis of WT (GH-WT) and *FmSPL2* transgenic tobacco (GH-919) plants using the RNA-seq method after drought treatment. Compared with GH-WT, a total of 3,703 genes exhibited differential expression in transgenic tobacco plants (GH-919), including 1,716 upregulated genes and 1,987 downregulated genes (Fig. 8a). Gene ontology (GO) annotation of these differentially expressed genes was performed, and the genes were assigned to the three biological processes of molecular function, cellular component, and biological process (Fig. 8b). Furthermore, KEGG pathway analysis showed that the differentially expressed genes were significantly clustered in plant hormone signal transduction, starch and sucrose metabolism, and phenylpropanoid biosynthesis (Fig. 8c).

**Figure 9:**
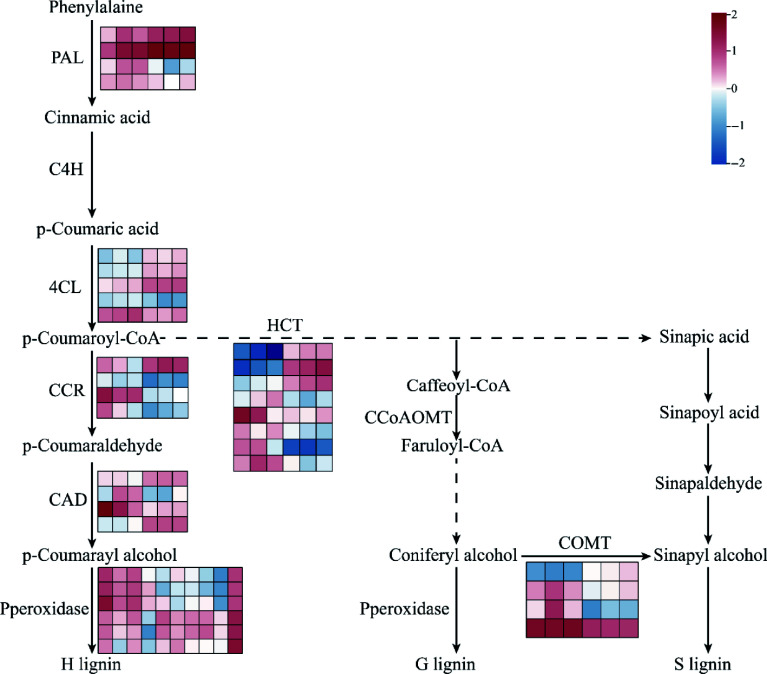
Phenylpropanoid and flavonoid biosynthesis network diagram. Gene expression is shown according to the mean FPKM of three biological replicates; blue indicates low expression and red indicates high expression. PAL, phenylalanine ammonialyase; C4H, cinnamate 4-hydroxylase; 4CL, 4-coumaroyl CoA ligase; CCR, cinnamoyl-CoA reductase; HCT, shikimate O-hydroxycinnamoyltransferase; CAD, cinnamyl-alcohol dehydrogenase; CCoAOMT, caffeoyl-CoA O-methyltransferase; COMT caffeic acid 3-O-methyltransferase.

Through transcriptomic analysis of *FmSPL2*-OE1 and WT plants under drought stress, we found that differentially expressed genes were primarily concentrated in phenylpropanoid metabolism. Additionally, the lignin content in *FmSPL2*-OE1 increased after drought stress. Therefore, our study focuses on the phenylpropanoid metabolism pathway. Phenylalanine, serving as a precursor for phenylpropanoid biosynthesis, is produced via the shikimate pathway, which constitutes a phenylpropane pathway activated in response to early abiotic stress and regulated by key rate-limiting enzymes PAL and 4CL. After drought induction, the expression levels of two *PAL* genes (*Nbe14g05190*, *Nbe08g14430*) and three *4CL* genes (*Nbe05g21820*, *Nbe06g22230*, *Nbe05g24290*) were significantly upregulated. In particular, within the phenylpropanoid biosynthesis pathway, HCT was the key fulcrum for the synthesis of H-, G-, and S-lignin, and determines the carbon source flow direction of lignin units^[34,35]^. Notably, three *HCT* genes (*Nbe06g00350*, *Nbe19g39960*, *Nbe09g00810*) exhibited enhanced expression in response to drought stress. COMT can catalyze the methylation of caffeic acid to synthesize ferulic acid, and then promotes the accumulation of sinapic acid. One *COMT* was upregulated, and three *COMT* were decreased under drought stress. CAD catalyzes the generation of *ρ*-coumaryl alcohol, with two genes (*Nbe17g19560*, *Nbe14g29220*) upregulated and two genes (*Nbe04g04970*, *Nbe03g09090*) downregulated. Lignin monomers, synthesized in the cytoplasm and polymerized within the cell wall, undergo final polymerization catalyzed by peroxidases, which are key enzymes determining lignin polymer structure. After drought stress, the expression of four peroxidase genes (*Nbe11g24490*, *Nbe05g32950*, *Nbe03g35800*, *Nbe19g21300*) were upregulated (Fig. 9).

**Figure 10:**
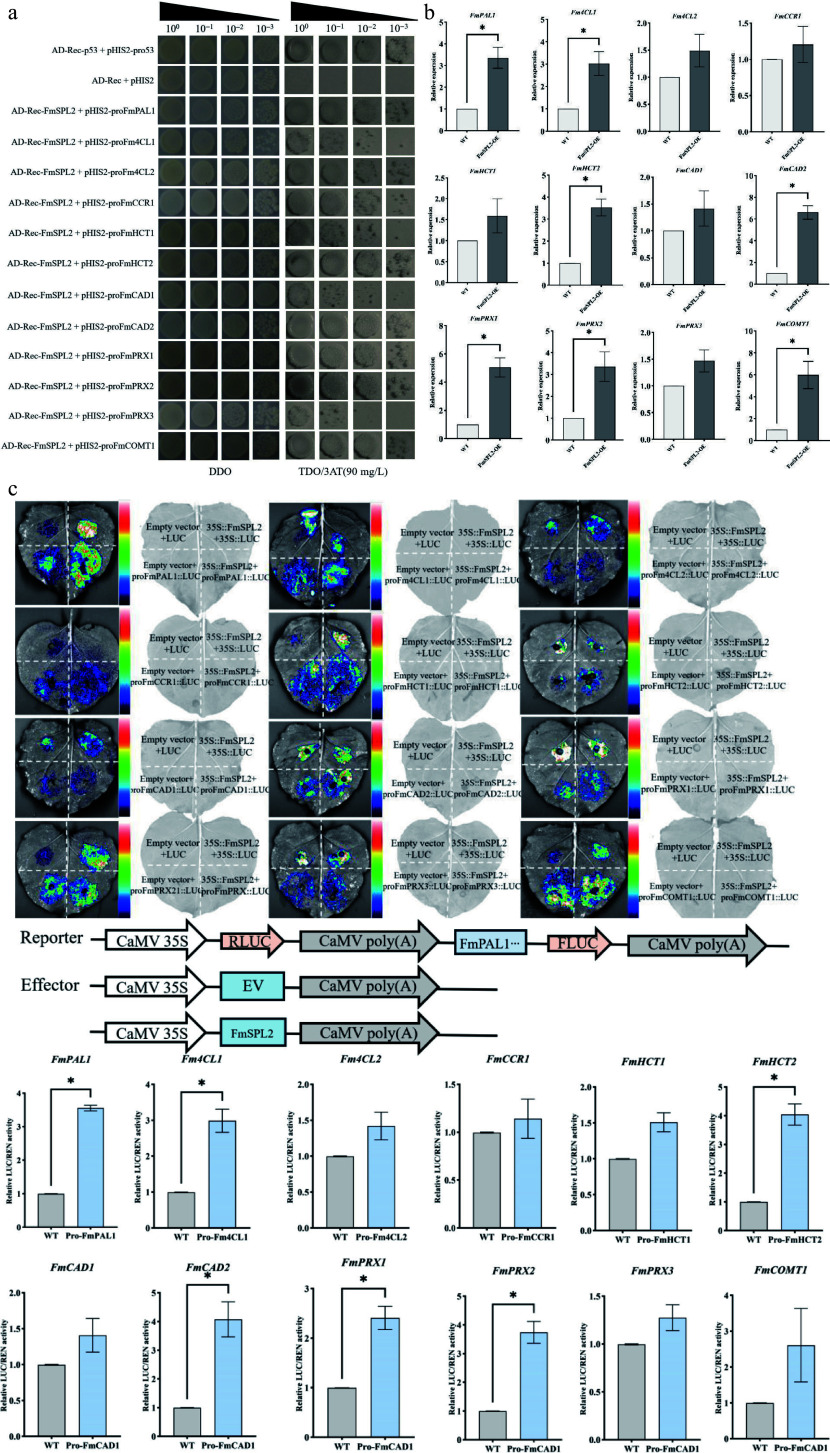
Verification of the binding and regulatory relationship between FmSPL2 and downstream lignin biosynthetic genes. (a) Yeast one-hybrid (Y1H) assay showing the physical binding of FmSPL2 to the promoters of genes related to the phenylpropanoid biosynthetic pathway. (b) Relative expression levels (qRT-PCR) of the target genes in *FmSPL2* overexpressing lines. (c) Dual-luciferase reporter assay in *N. benthamiana* leaves demonstrating the transcriptional activation of downstream gene promoters by FmSPL2.

To further validate the role of *FmSPL2* in regulating downstream target genes in *Fraxinus mandshurica*, we performed a qPCR experiment after transient transformation and PEG-simulated drought treatment to verify the regulatory relationship. The qPCR results showed that, after transient transformation of *FmSPL2* and drought treatment, the expression of genes such as *PAL1*, *4CL1*, and *PRX2* was upregulated, thereby confirming the reliability of the transcriptome data (Fig. 10b). Furthermore, we analyzed the promoter regions of all upregulated downstream genes in *Fraxinus mandshurica* and found that their upstream 2,000 bp sequences contained the conserved SBP binding motif GTAC. To verify the regulatory relationship, yeast one-hybrid (Y1H) and dual-luciferase reporter assays were performed. The yeast one-hybrid assay confirmed that FmSPL2 can bind to the promoters of *FmPAL*, *Fm4CL*, *FmHCT*, *FmCAD*, and *FmPRX* (Fig. 10a). This was further supported by the dual-luciferase reporter assay in *N. benthamiana* leaves, which demonstrated that FmSPL2 significantly activated the transcription of these downstream genes (Fig. 10c). Furthermore, the assay indicated that FmSPL2 enhances drought resistance in *Fraxinus mandshurica* by regulating the expression of genes involved in the phenylpropanoid metabolism pathway.

## Discussion

In recent years, the frequency and intensity of drought events have significantly increased, thereby emerging as a critical factor affecting the stability of global ecosystems^[36]^. Drought severely affects plant growth by altering water distribution, and biochemical and physiological parameters, thereby prompting plants to evolve various defense mechanisms to enhance survival^[37]^. Investigating the molecular mechanisms underlying plant adaptation to abiotic stress is essential for elucidating how plants respond to environmental fluctuations and provides a theoretical basis for the development of novel high-yield, drought-resistant germplasm with improved environmental adaptability. Among these, the SPL transcription factor family, which is unique to green plants, plays a pivotal role in modulating plant growth and development as well as in plant stress defense responses. In *Fraxinus mandshurica*, 36 *SPL* genes have been identified, with *FmSPL1*, *FmSPL2*, *FmSPL3*, *FmSPL7*, *FmSPL8*, *FmSPL9*, *FmSPL10*, and *FmSPL12* exhibiting responsiveness to NaCl, low temperature, and hormone treatments^[33]^. Furthermore, overexpression of *FmSPL2* in transgenic *Nicotiana tabacum* results in taller plants with shorter roots, increased root numbers, rounder leaves, and earlier flowering^[33]^. However, its role in drought adaptation remains unclear. Previous research has demonstrated that phenylpropanoid metabolism is a crucial metabolic pathway involved in maintaining homeostasis in plants under environmental stress^[38]^. The present study investigates the role of FmSPL2 in augmenting drought resistance by regulating the expression of key enzymes within the phenylpropanoid metabolic pathway, thereby promoting lignin biosynthesis.

### *FmSPL2* enhances drought resistance by activating antioxidant defense, regulating stomatal aperture, and photosynthesis

Drought stress induces substantial ROS production in plants, primarily as a consequence of inhibited carbon fixation resulting from stomatal closure, which subsequently impairs photosynthetic processes^[39]^. As shown in Fig. 1a, the expression of *FmSPL2* was upregulated following 3 h of drought treatment, concomitantly promoting the expression of drought-related genes *SnRK2.6*^[40]^ and *AREB1*^[41]^, indicating that *FmSPL2* responds to drought stress. To further explore how *FmSPL2* affects plant drought resistance, we observed the phenotypic characteristics and assessed physiological parameters in tobacco plants with stable *FmSPL2* overexpression (Fig. 3) and *Fraxinus mandshurica* subjected to VIGS silencing of *FmSPL2* (Fig. 2). We found that *FmSPL2* enhances drought resistance through the regulation of ROS homeostasis. Previous studies have shown that SOD enzymes constitute the primary defense mechanism against ROS by catalyzing the dismutation of superoxide radicals, thereby augmenting plant stress tolerance^[42]^. Additionally, POD enzymes reduce H_2_O_2_ to alleviate oxidative damage caused by stress^[43]^. After drought stress, ROS accumulation was significantly reduced in *FmSPL2* overexpressing plants relative to WT plants (Fig. 6), while VIGS plants exhibited the opposite result (Fig. 2). Hanly et al. found that RNAi silencing of the transcription factor *MsSPL9* in transgenic alfalfa led to upregulation of *MsCAT1* and *MsGSH* expression compared to WT *Medicago sativa*, alongside enhanced anthocyanin biosynthesis, collectively contributing to improved drought tolerance^[44]^. Similarly, research on *Manihot esculenta* showed that the transcription factor MeSPL9 regulates cassava's drought resistance by modulating JA signaling and antioxidant levels^[45]^. The antioxidant defense system in plants effectively manages ROS and maintains intracellular redox homeostasis^[46]^. Specifically, antioxidant enzymes alleviate drought-induced lipid peroxidation by scavenging superoxide radicals, thereby protecting plants from damage. In our study, *FmSPL2* plays a crucial role in drought adaptation by activating the ROS scavenging system.

Under drought conditions, stomatal conductance decreases, leading to a reduced diffusion of carbon dioxide through the stomata and mesophyll, which lowers the carbon dioxide concentration in photosynthetic cells and inhibits carbon fixation. Concurrently, diminished water availability leads to decreased leaf cohesion, which further suppresses photosynthesis and long-distance transport of substances^[47,48]^. After drought stress, the stomatal conductance in *FmSPL2*-OE1 and *FmSPL2*-OE2 was significantly lower than that of the WT, indicating that *FmSPL2* enhances drought resistance by reducing stomatal aperture and reducing transpiration rate (Fig. 4). Although an increase in stomatal density was detected in the transgenic lines compared to the WT, the stomatal aperture was significantly reduced. Generally, increased stomatal density is associated with higher water loss; however, the simultaneous decrease in aperture observed here suggests a coordinated compensatory mechanism. This adaptive plasticity enables the plant to respond more sensitively to water deficit, effectively limiting transpiration and enhancing water use efficiency (WUE) despite the higher number of stomata^[49,50]^. Chlorophyll fluorescence serves as a rapid method to estimate the quantum efficiency of electron transport through PSII in leaves, thereby facilitating the monitoring of photosynthetic performance^[51]^. The chlorophyll fluorescence parameters F_v_/F_m_ and F_v_/F_0_ are indicative of the photosynthetic efficiency and activity of PSII, respectively^[52]^. When the light energy absorbed by plants exceeds the light energy required for photosynthesis, photoinhibition occurs, and a decrease in the F_v_/F_m_ ratio is commonly considered a marker of photoinhibition^[53]^. After drought stress, we observed that the F_v_/F_m_ ratio of WT was lower than that of *FmSPL2* transgenic tobacco (Fig. 5), suggesting that drought caused more severe damage to WT tobacco. A study on rice found that *OsSPL10* regulates stomatal closure under drought stress, affecting the water loss rate and subsequently influencing drought resistance^[27]^.

### *FmSPL2* enhances lignin synthesis to improve drought resistance

Lignin is the second most abundant complex phenolic compound in nature and the primary constituent of plant cell walls^[5]^. Its biosynthetic pathway involves a range of key functional enzymes and is regulated by various transcription factors. Among these, the *SPL* gene family plays an important role in the regulation of lignin synthesis, thereby affecting vascular tissue development. For example, research conducted on *Arabidopsis thaliana* has demonstrated that miR857 is specifically expressed within vascular tissues. Under low copper conditions, miR857 expression is induced by *SPL7*, which contributes to the regulation of lignin content in the secondary xylem cell wall, demonstrating the regulatory function of *AtSPL7* in the secondary growth of vascular tissues in *Arabidopsis thaliana*^[54]^. In addition, a total of 35 full-length *SPL* genes have been identified in the genome of *Panicum virgatum*. Among these, *PvSPL2* regulates gene expression by directly binding to the promoters of the three lignin biosynthesis genes *CCR1*, *F5H*, and *COMT*, thereby affecting lignin biosynthesis^[28]^. In *OsSPL14* transgenic rice plants, the transcriptional levels of *C4H*, *4CL*, and *PAL* are upregulated, leading to a significant increase in lignin and cellulose content in the stems. This biochemical enhancement subsequently improves the mechanical strength and lodging resistance of the *OsSPL14* transgenic plants^[29]^.

**Figure 11:**
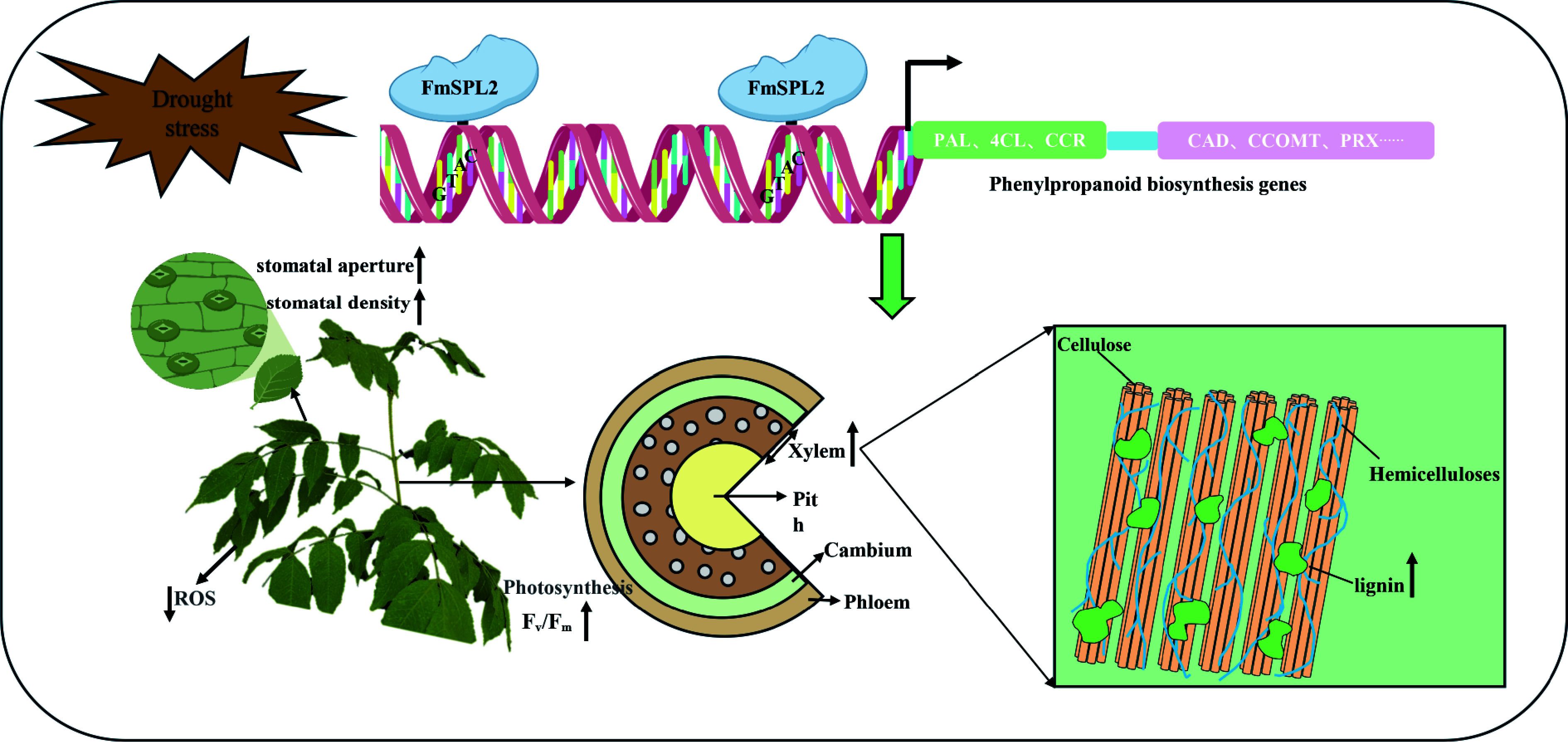
Proposed molecular model demonstrating that *FmSPL2* regulates lignin biosynthesis and drought adaptation in *Fraxinus mandshurica*. *FmSPL2* is rapidly expressed under drought stress, regulating the expression of phenylpropanoid metabolism-related genes to promote lignin biosynthesis. At the same time, it enhances drought tolerance in *Fraxinus mandshurica* by modulating stomatal aperture, improving photosynthetic efficiency, and increasing ROS scavenging capacity.

Moreover, a substantial body of research indicates that the phenylpropanoid pathway plays a crucial role in improving drought tolerance in plants^[38]^. In this study, before drought treatment, compared to the WT, the xylem of *FmSPL2-OE* plants was thicker, especially at the sixth internode, where the xylem of *FmSPL2*-OE was 26% thicker than that of the WT. Lignin content analysis showed that under normal conditions, the lignin content in *FmSPL2*-OE was 19.87% and 18.48% higher than in WT. After drought treatment, the lignin content in *FmSPL2*-OE1 and *FmSPL2*-OE2 was 17.23% and 18.26% higher, respectively, compared to WT. To further demonstrate that *FmSPL2* can regulate lignin biosynthesis after drought stress, we performed RNA-seq analysis on *FmSPL2*-OE and WT plants subjected to drought stress. KEGG analysis revealed that differentially expressed genes were enriched in the phenylpropanoid biosynthesis pathway, and we found a significant increase in the expression of phenylpropanoid pathway genes such as *PAL*, *CAD*, and *4CL* in *FmSPL2*-OE plants after drought treatment (Fig. 9). To further confirm that FmSPL2 can regulate lignin biosynthesis gene expression in *Fraxinus mandshurica*, we transiently transformed the species and applied PEG to simulate drought stress. qPCR results showed upregulation of genes such as *PAL*, *4CL*, and *HCT*. We also cloned the 2 kb upstream promoter sequences of these genes. Yeast one-hybrid and dual-luciferase reporter assays indicated that *FmSPL2* could bind to the promoter regions of *PAL*, *4CL*, and *PRX*, thereby activating the expression of these genes. Therefore, phenylpropanoid metabolism increases with enhanced drought resistance in *Fraxinus mandshurica*. The increase in lignin in *Fraxinus mandshurica* under drought promotes the lignification of cell walls and reduces cellular water loss (Fig. 11).

## Conclusions

This study used VIGS-silenced FmSPL2 *Fraxinus mandshurica* plants and *FmSPL2* transgenic tobacco plants as materials and analyzed their physiological characteristics and transcriptomic regulation after drought treatment. The results showed that *FmSPL2* enhances drought resistance by increasing antioxidant enzyme activity against ROS, reducing stomatal conductance to decrease water transpiration, enhancing photosynthetic rate to maintain carbon assimilation capacity, and promoting lignin biosynthesis by regulating the expression of key enzyme genes like *PAL*, *CAD*, and *PRX* in the phenylpropanoid metabolism pathway. These findings elucidate the molecular regulatory mechanisms of *FmSPL2* in drought adaptation and xylem development, providing a theoretical basis for the genetic breeding of *Fraxinus mandshurica*. They also have important practical implications for improving wood quality traits and enhancing stress resistance.

## SUPPLEMENTARY DATA

Supplementary data to this article can be found online.

## Data Availability

The RNA-seq data underlying this article is available in the Genome Sequence Archive (GSA) at https://ngdc.cncb.ac.cn and can be accessed with CRA038042.
