# Supplementary material for: Fraxinus mandshurica SPL2 enhances tolerance to drought stress by activating the phenylpropanoid pathway
Source: For Res (Fayettev). 2026 Apr 14;6:e015. doi: 10.48130/forres-0026-0015 (PMC13195492; doi:10.48130/forres-0026-0015)
Supplement: Supplementary file 1 — Supplementary data to this article can be found online. [file forres-0026-0015-S1.zip › 10.48130_forres-0026-0015-Suppl-FigureS1.pdf]

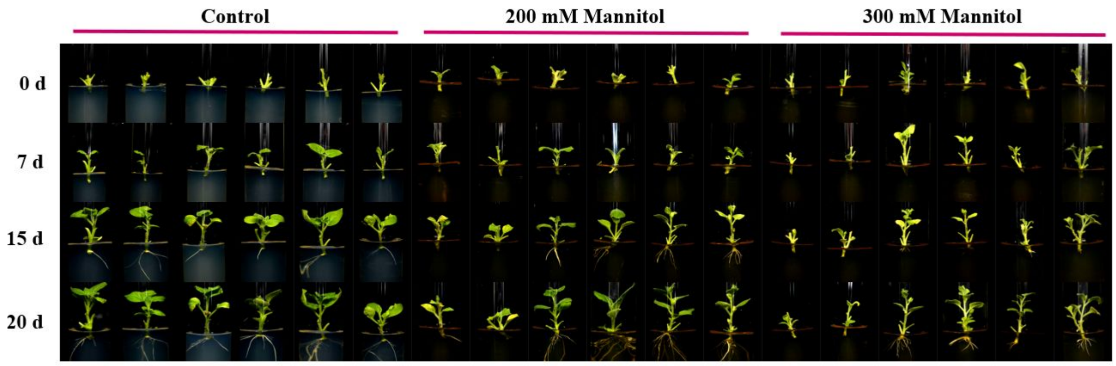

Fig. S1 *FmSPL2* transgenic tobacco simulated drought treatment phenotype image. *In vitro* culture with 200 mM and 300 mM mannitol to simulate drought treatment.
